# Etiology and the challenge of diagnostic testing of community-acquired pneumonia in children and adolescents

**DOI:** 10.1186/s12887-022-03235-z

**Published:** 2022-03-31

**Authors:** Zulma Vanessa Rueda, Yudy Aguilar, María Angélica Maya, Lucelly López, Andrea Restrepo, Carlos Garcés, Olga Morales, Claudia Roya-Pabón, Mónica Trujillo, Catalina Arango, Ángela Rocio Copete, Cristian Vera, Margarita Rosa Giraldo, Mariana Herrera, Lázaro A. Vélez

**Affiliations:** 1 Department of Medical Microbiology and Infectious Diseases, University of Manitoba, Winnipeg, Canada; 2 Grupo Investigador de Problemas en Enfermedades Infecciosas (GRIPE), Facultad de Medicina, Universidad de Antioquia UdeA, Medellín, Colombia; 3 Clínica Universitaria Bolivariana, Universidad Pontificia Bolivariana, Medellín, Colombia; 4 Unidad de Enfermedades Infecciosas, Hospital Universitario San Vicente Fundación, Medellín, Colombia; 5 Facultad de Medicina, Universidad Pontificia Bolivariana, Medellin, Colombia; 6 Departamento de Pediatría, Hospital Pablo Tobón Uribe, Medellín, Colombia; 7 Departamento de Pediatría, Universidad CES, Medellín, Colombia; 8 Departamento de Pediatría y Puericultura, Grupo Pediaciencias, Universidad de Antioquia UdeA, Medellín, Colombia; 9 Departamento de Pediatría, Hospital Universitario San Vicente Fundación, Medellín, Colombia; 10 Tuberculosis Clinic, Pima County Health Department, Tucson, USA; 11 Laboratorio Integrado de Medicina Especializada, Universidad de Antioquia UdeA, IPS Universitaria, Medellin, Colombia; 12 Secretaría Seccional de Salud y Protección Social de Antioquia, Gobernación de Antioquia, Medellín, Colombia

**Keywords:** Pneumonia, Diagnosis, Etiology, Multiplex PCR, Serology, Induced sputum, Nasopharyngeal swab, Urine antigen, Children

## Abstract

**Background:**

Pneumonia is the leading cause of mortality in pediatric population. The etiology of pneumonia in this population is variable and changes according to age and disease severity and where the study is conducted. Our aim was to determine the etiology of community-acquired pneumonia (CAP) in children aged 1 month to 17 years admitted to 13 Colombian hospitals.

**Methods:**

Prospective cohort study. Hospitalized children with radiologically confirmed CAP and ≤ 15 days of symptoms were included and followed together with a control group. Induced sputum (IS) was submitted for stains and cultures for pyogenic bacteria and *Mycobacterium tuberculosis*, and multiplex PCR (mPCR) for bacteria and viruses; urinary antigens for pneumococcus and *Legionella pneumophila*; nasopharyngeal swabs for viruses, and paired serology for atypical bacteria and viruses. Additional cultures were taken at the discretion of primary care pediatricians.

**Results:**

Among 525 children with CAP, 71.6% had non-severe pneumonia; 24.8% severe and 3.6% very severe pneumonia, and no fatal cases. At least one microorganism was identified in 84% of children and 61% were of mixed etiology; 72% had at least one respiratory virus, 28% pyogenic bacteria and 21% atypical bacteria. Respiratory syncytial virus, Parainfluenza, Rhinovirus, Influenza, *Mycoplasma pneumoniae*, Adenovirus and *Streptococcus pneumoniae* were the most common etiologies of CAP. Respiratory syncytial virus was more frequent in children under 2 years and in severe pneumonia. Tuberculosis was diagnosed in 2.3% of children. IS was the most useful specimen to identify the etiology (33.6%), and blood cultures were positive in 3.6%. The concordance between all available diagnostic tests was low. A high percentage of healthy children were colonized by *S. pneumoniae* and *Haemophilus influenzae*, or were infected by Parainfluenza, Rhinovirus, Influenza and Adenovirus.

**Conclusions:**

Respiratory viruses are the most frequent etiology of CAP in children and adolescents, in particular in those under 5 years. This study shows the challenges in making an etiologic diagnosis of CAP in pediatric population because of the poor concordance between tests and the high percentage of multiple microorganisms in healthy children. IS is useful for CAP diagnosis in pediatric population.

**Supplementary Information:**

The online version contains supplementary material available at 10.1186/s12887-022-03235-z.

## Background

Community-acquired pneumonia (CAP) is the leading cause of mortality in children under 5 years of age in low- and middle-income countries [1]. It is estimated than lower respiratory tract infections caused 2,377,697 deaths worldwide in 2016, of which 652,572 occurred in children under 5 years of age. The highest proportion occurred in the Sub-Saharan Africa region (27.4%), followed by South Asia (24.8%); Latin America and the Caribbean contributed 6.8% [2–5]. In Colombia in 2018, the mortality rate due to acute respiratory infections was 14.89 per 100,000 children under 5 years of age [6].

The annual global incidence of CAP is close to 0.22 episodes per child per year or 155 million new cases per year in the world, of which 10–17% require hospitalization [5]. A study showed that the incidence of pneumonia-related hospitalization in the United States was 15.7 cases per 10,000 children per year but varies according to age group, from 62.2/10,000 in those under 2 years of age to 4.2/10,000 in the group of 10–17 years old [7].

The etiology of pneumonia in the pediatric population is variable and changes according to age and disease severity and where the study is conducted [7]. Microbiological identification is challenging, since collection of respiratory samples in children is problematic in comparison to adults [8]. Some microbiological tests have low sensitivities in children. Previous research has shown different etiologic attributions according to the anatomic origin of collected specimens (induced sputum, nasopharyngeal or oropharyngeal aspirate or swab, blood and/or urine) and the diagnostic tests employed (PCR, acute or convalescent serology, cultures, direct or indirect immunofluorescence) [9].

Molecular techniques have enabled identification of the likely etiology in up to 86% of pediatric CAP cases [10, 11]. These techniques have also detected mixed infection as the cause of pneumonia, ranging from 23.7% in Asia to 33.8% in Europe [12]. However, one of the challenges to interpret the results of those tests is that the microorganisms causing pneumonia are commonly commensal flora instead of real pathogens.

The broad uptake of pneumococcal and *Haemophilus influenzae* type B (Hib) vaccines in many countries have reduced the number of pneumonia cases and hospitalizations in children and changed the etiology [13–18]. In 2010, the expanded immunization plan in Colombia included pneumococcal vaccine (PCV10) for children under 2 years of age [6]. A study of CAP etiology in children younger than 18 years in the United States, in the era of access to pneumococcal and Hib vaccination, showed that viruses and *Mycoplasma pneumoniae* accounted for the first eight causes, and the ninth cause was *S. pneumoniae* [7].

Studies of pediatric CAP etiology in middle-income countries are required in order to update treatment and vaccination guidelines and to promote the development of diagnostic tests that identify the most common microorganisms causative of pneumonia. We aimed to determine the etiology of CAP in hospitalized children and adolescents between 1 month and 17 years in Antioquia, Colombia**,** and to evaluate the utility of different specimens as well as diagnostic tests available in the country.

## Materials and methods

### Study design

Prospective cohort study between August 2011 and September 2012.

### Setting

Thirteen hospitals of medium and high complexity care from Medellin and three nearby cities (Itagui, Bello, Envigado) were included.

### Sample size

CAP group: According to national statistics from 2007, there were 1595 children hospitalized with pneumonia in these four cities. We took into consideration the respiratory pathogen with the lower percentage for the estimation of the simple size: 4% of Bocavirus [19]. With an error of 1.5% and a 95% confidence interval, the estimated sample was 465 children. The sample size was increased by 10.5% to account for lost-to follow-up, for a final sample size of 512.

Control group: A convenience sample of 61 healthy children and adolescents were chosen as a control group. The number of persons in the control group was based on budgetary constraints. This group was recruited with the objective to explore the presence of respiratory bacteria and viruses that we would evaluate in pediatric population with CAP among healthy children and adolescents.

### Inclusion criteria

CAP group: We included children and adolescents between 1 month and 17 years of age with CAP that required hospitalization, with respiratory symptoms less than 15 days and for whom a guardian or legal representative and the child agreed to participate. CAP was defined as the presence of any alveolar or interstitial opacity in the chest x-rays plus one of the following symptoms or signs: i) axillary fever ≥38.3 °C; ii) tachypnea (Children < 2 months: ≥60/min; between 2 and 11 months: ≥50/min; 1–4 years: ≥40/min; 5–12 years: > 30/min; > 12 years: > 20/min), and iii) the presence of rhonchi and/or crackles and/or wheezing.

Control group: Children and adolescents between 1 month and 17 years old, resident in Medellín, who underwent ambulatory elective surgery for unrelated causes and had no CAP or any respiratory infection in the last month prior to the elective surgery and required endotracheal intubation. Written informed consent and assent when indicated was obtained from their parents, caregivers and themselves.

### Exclusion criteria

CAP group: Previous hospitalization in the last 15 days; antibiotic treatment for more than 72 h at the time of hospital admission; primary or severe acquired immunodeficiency; cystic fibrosis; bronchiolitis obliterans; neurological (cerebral palsy) or neuromuscular disorders, or psychiatric disorders that did not allow the patient to assent; congenital metabolic disorders; bronchiolitis (first sibilant episode in children less than 2 years); hematological malignancies; neutropenia < 500 cells/mm^3^; primary ciliary dyskinesia; AIDS, or CD4 cell count less than 15% in HIV-infected children 5 years or younger, or CD4 cell count < 200 cells/mm^3^ in HIV-infected children older than 5 years; treatment with prednisolone ≥1 mg/kg/day or its equivalent for more than 8 days, or other immunosuppressive drugs such as cyclosporine, methotrexate, mycophenolate mofetil, cyclophosphamide, azathioprine and fluorouracil.

Control group: None, apart from fulfilment of inclusion criteria.

### Procedures and specimen collection

CAP group: A nasopharyngeal swab (NS) was collected with a nylon swab (Copan Flocked Swabs®) and transferred to 1 ml vials containing normal saline. One induced sputum (IS), urine and whole blood samples were collected, the latter during enrollment (acute phase) and 4 to 12 weeks after (convalescent phase). In addition, a NS was collected for detection of *Bordetella pertussis*. IS was obtained after inhalation of β-2 agonist (200 μg) and nebulization of 5% hypertonic saline; once the children had productive cough, we collected the IS into the container, or respiratory secretions were aspirated into a Lukens trap using a suction catheter (Fr 6–8) that was introduced into the oropharynx via the nasopharynx [20]. The limit for introducing the nasopharyngeal swab was the midpoint between the tip of the nose and the tragus of the ear. The IS sample was divided into 3 samples: one for Gram stain and aerobic culture, a second for detection of *Mycobacterium tuberculosis* by stain and culture*,* and a third sample for multiplex PCR (mPCR) for bacteria and viruses. *A trained nurse* administered the tuberculin skin test (tuberculin PPD RT-23, 2 T.U/0.1 ml, Statens Serum Institut®) to all children that accepted, according to CDC guidelines [21]. Reading was performed 48 to 72 h later and measured in millimeters of induration.

Control group: After endotracheal intubation, NS, blood, and urine samples were obtained. A tracheal aspirate was obtained for mPCR for bacteria and viruses. For this procedure, 10 ml of normal saline 0.9% were instilled, then suctioned with a catheter and collected with a Lukens trap.

Transport and processing of specimens: Immediately after collection, 1 ml of RNA Later® Solution (Ambion® Life Technologies) was added to the IS and tracheal aspirate samples to reduce the RNA degradation process. The samples were transported at 2–8 °C to the laboratory. Urine samples were processed upon arrival. Serum samples were stored at − 20 °C and the IS, NS and tracheal samples were stored at − 80 °C until processing.

### Microbiologic studies

Gram stain and cultures: In IS, Gram stain was performed to determine the quality of the sputum sample; only those with < 25 squamous epithelial cells (SECs)/per low-power field (LPF) were cultured and inoculated onto blood, chocolate and MacConkey agars. Blood cultures were obtained in all children, except when the primary care physician considered them not indicated. When available, pleural fluid was collected and was also processed for microbiology test (Gram stain, culture and according to pediatrician’s criteria, mycobacterium culture).

Serology: Paired serum samples in acute and convalescent phases were tested for atypical bacteria (*Mycoplasma pneumoniae*, *Chlamydophila pneumoniae*, *Legionella pneumophila*, and *Coxiella burnetii*) and conventional respiratory viruses (Adenovirus, Influenza A and B, Parainfluenza 1, 2, 3 and Respiratory Syncytial Virus). IgG and IgM antibodies of *C. pneumoniae* were assayed using micro-IFA (Indirect Immunofluorescence Assay) (Focus Diagnostics®, Cypress, California); IgG and IgM antibodies to phase I and II of C. burnetii by IFA (Focus Diagnostics®); and total antibodies against *L. pneumophila by *IFA (Focus Diagnostics®); and IgG and IgM antibodies against* M. pneumoniae* were determined by immunoenzymatic assay (Vircell®, Granada, Spain). In addition, *Chlamydia trachomatis *IgM was detected in acute serum sample by micro-IFA (Focus Diagnostics®)*.* Immunologic responses to respiratory viruses were detected semiquantitavely by immunoenzymatic assays (Adenovirus IgG; Influenza A/B IgG; Parainfluenza 1, 2,3 IgG; RSV IgG; RIDASCREEN®, Darmstadt, Germany). At the beginning of the study the assay available did not discriminate between Influenzae A or B, but afterwards they were detected separately.

Urine antigens: Detection of *S. pneumoniae and L. pneumophila *serogroup 1antigens was done in centrifuged urine samples (at 2500 rpm for 10 min) using the *Alere BinaxNOW® Legionella and Streptococcus pneumoniae *kits*.*

Viral and bacterial antigens: In NS, Adenovirus, Influenza A, Influenza B, Parainfluenza type 1, 2 and 3 and RSV were detected by indirect immunofluorescence (Respiratory Panel 1 Viral Screening & Identification Kit, LIGHT DIAGNOSTICS™)*.* Direct immunofluorescence was done in nasopharyngeal and conjunctival samples for *B. pertussis and C. trachomatis*.

Studies for *Mycobacterium tuberculosis: *In IS samples auramine-rodamine stain and cultures solid* (*Lowenstein-Jensen and thin-layer agar) and liquid (BD Bactec™ MGIT™ 960) media were done.

### Nucleic acid extraction

IS samples (500 μL) were treated with N-acetyl cysteine and 2% NaOH for 15 min at room temperature. The DNA extractions were performed with QIAmp DNA (Qiagen, Valencia, CA, USA) and with NucleoSpin®RNA Virus for RNA (Macherey-NAgel, Düren, Germany).

### Multiplex PCR for bacteria and viruses

The mPCR Seeplex® RV12 or RV15 ACE Detection (Seegene, Inc.) were used for detection of 16 respiratory viruses (human adenovirus (AdV), human coronavirus 229E, NL63, OC43, human parainfluenza 1 (PIV1), 2 (PIV2) and 3 (PIV3), human rhinovirus A/B/C (HRV), respiratory syncytial virus A (RSVA) and B (RSVB), influenza A (FluA) and B (Flu B), bocavirus 1/2/3/4 (HBoV), human Metapneumovirus (MPV), parainfluenza 4 (PIV4) and human enterovirus (HEV)). In addition, six bacteria were detected (*M. pneumoniae, L. pneumophila, S. pneumoniae, H. influenzae, B. pertussis* and *C. pneumoniae*), using the mPCR Seeplex® Pneumobacter ACE Detection.

Both mPCR assays were processed with an internal control, a positive (plasmids of the 6 bacteria or the 15 respiratory viruses, accordingly) and a negative control. Finally, a conventional PCR to amplify GADPH was done to verify the presence of human genetic material in all clinical samples.

All conventional and molecular protocols were done following the instructions of the manufacturers. CLSI and European guidelines for molecular diagnostics were also followed.

### Variables and definitions

All microbiological diagnosis definitions are reported in Appendix 1.

We collected sociodemographic and clinical variables, including age, sex, vaccination record; age-appropriate vaccination was defined according to Colombian immunization program at that time [22]. Comorbidities, prior history of antibiotics (in the last 3 months and within the last 48 h) and hospitalization within the last year, symptoms, complications during hospitalization (pleural effusion, lung abscess, empyema, acute renal disease, acute respiratory distress syndrome and admission to intensive care unit), length of stay and mortality.

Severity of pneumonia was defined according to the World Health Organization (WHO) classification of childhood pneumonia: 1) Non-severe (pneumonia): fast breathing; 2) severe: chest indrawing; and 3) Very severe: not able to drink, persistent vomiting, convulsions, lethargic or unconscious, stridor in a calm child, or severe malnutrition [23].

### Statistical methods

Descriptive statistics were used for clinical variables. The frequencies for each microorganism and diagnostic tests employed were estimated for CAP and control groups**.** The distributions of microorganisms according to age group and the severity of CAP are described.

## Results

### Population

**Fig. 1:**
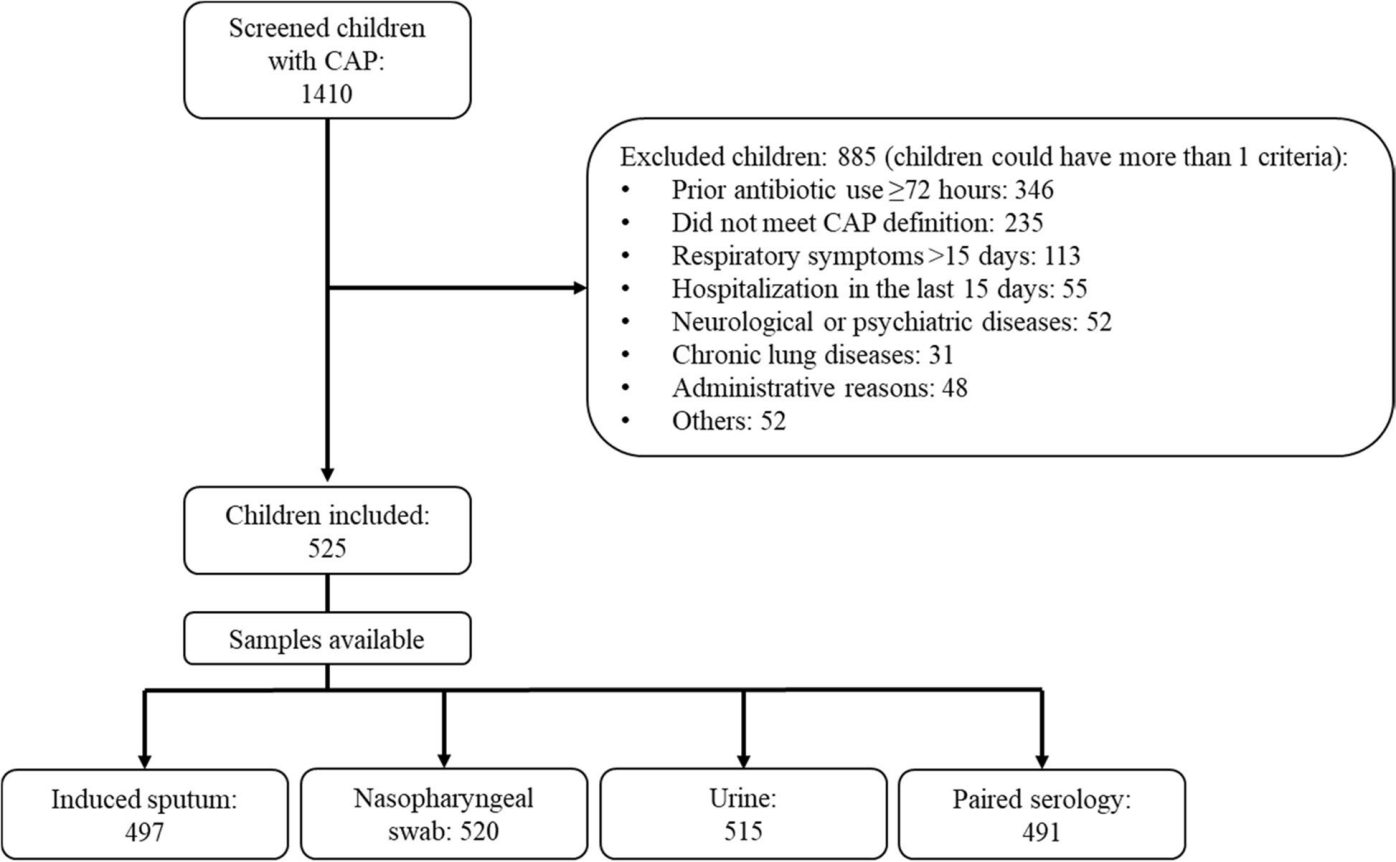
Children and adolescents with community-acquired pneumonia enrollment flowchart

We screened 1410 children and 885 were excluded. The main reason for exclusion was antibiotic consumption within the 72 h previous to admission (Fig. 1); 525 children and adolescents were included. Among the control group, 74 children were evaluated, and 61 enrolled. The adjusted annual incidence of childhood CAP in Medellín (younger than 18 years who need hospitalization) was 147/100,000 (608/100,000 from 1 to 23 months, 299/100,000 in those from 2 to 4 years, and 42/100,000 between 5 to 17 years).

### Characteristics of CAP and control group

Among the CAP group, around 80% were ≤ 5 years of age, a quarter had received antibiotics in the last 3 months, and 26% had been previously hospitalized for pneumonia. Most children in both groups were living in urban areas and had age-appropriate vaccination (Table 1).

**Table 1:** Baseline characteristics of 525 children and adolescents with community acquired pneumonia and 61 controls

Variables	CAP group (*N* = 525)	Control group (*N* = 61)
Age in months, median (IQR)	32 (14–53)	53 (29–72)
	n/N (%)	
Age by groups		
0–23 months	206/525 (39.2)	12/61 (19.7)
2–4 years	213/525 (40.6)	23/61 (37.7)
≥5 years	106/525 (20.2)	26/61 (42.6)
Male	267/525 (50.9)	44/61 (72.1)
Urban area	401/525 (76.4)	61/61 (100)
Vaccination record (according to card available)	417/525 (79.4)	25/61 (41.0)
Pentavalent vaccine (DPT, Hep B, Hib)	400/415 (96.4)	25/25 (100)
Fully vaccinated for age	375/404 (92.8)	23/25 (92.0)
Pneumococcal vaccine^a^	197/416 (47.4)	16/25 (64.0)
0–23 months	107/173 (61.8)	6/16 (37.5)
2–4 years	73/176 (41.5)	6/16 (37.5)
≥5 years	17/67 (25.4)	4/16 (25.0)
Influenza vaccine	172/410 (42.0)	18/25 (72.0)
Any associated condition	154/523 (29.4)	ND
Asthma	139/523 (26.6)	–
Others^b^	23/523 (4.4)	–
History of prior antibiotics in the last 3 months	121/522 (23.2)	6/61 (9.8)
Prior antibiotics in the last 48 h	38/523 (7.3)	0 (0)
Prior hospitalization due to pneumonia	138/523 (26.4)	ND
< 3 months	21/138 (15.2)	–
3 months – 11 months	63/138 (45.7)	–
≥1 year	54/138 (39.1)	–

^a^Any pneumococcal vaccine (conjugated with 7, 10 or 13 serotypes, or non-conjugated with 23 serotypes)

^b^ Seizures (13), heart diseases (4), diabetes mellitus (2), chronic renal disease (1), collagen disease (1), pregnancy (1), HIV (1). ND: No data

Cough and fever were the most frequent symptoms (> 90%), followed by dyspnea (75%) and tachypnea (49%). Most cases were classified as non-severe, but 3.6% had very severe pneumonia, 6.1% had a complicated evolution, and 4.2% needed intensive care. There were no deaths during the study period (Table 2).

**Table 2:** Clinical characteristics and outcomes of children and adolescents with community acquired pneumonia

Variables	n/N (%)
Cough	513/525 (97.7)
Productive	322/513 (62.7)
Fever	485/525 (92.4)
Dyspnea	398/525 (75.8)
Tachypnea^a^	256/518 (49.4)
Irritability	240/525 (45.7)
Somnolence	214/525 (40.8)
Diarrhea	135/525 (25.7)
Earache	50/525 (9.5)
Oxygen saturation < 90% at the time of hospital admission	176/453 (38.9)
Severity of community acquired pneumonia	
Non severe	376/525 (71.6)
Severe	130/525 (24.8)
Very severe	19/525 (3.6)
Complications	32/524 (6.1)
Pleural effusion	22/524 (4.2)
Acute respiratory distress syndrome	13/524 (2.5)
Empyema	11/523 (2.1)
Lung abscess	3/524 (0.6)
Acute renal disease	1/524 (0.2)
Length of stay in days, median (IQR)	4 (3–6)
Admission to intensive care unit	22/524 (4.2)
Mortality	0/525 (0.0)

^a^ ≥60 breath/min for children younger than 2 months; ≥50 for children between 2 to 11 months; ≥40 children between 1 to 5 years; ≥30 between 5 to 12 years; and > 20 for children older than 12 years

### Etiology

Respiratory Syncytial Virus (RSV) was the most common cause of CAP (31.2%), followed by Parainfluenza, Rhinovirus, Influenza, *M. pneumoniae*, Adenovirus and *S. pneumoniae*. *B. pertussis* was recognized in 25 children, and tuberculosis was diagnosed in 12 cases, 6 confirmed by liquid culture (Table 3).

**Table 3:** Microbiologic diagnosis in 525 children and adolescents with community acquired pneumonia, stratified by age group

Respiratory pathogen	Age by groups			Total
	1–23 months *N* = 206 n (%)	2–4 years *N* = 213 n (%)	≥5 years *N* = 106 n (%)	n/525 (%)
Respiratory syncytial virus A and B	91 (44.2)	56 (26.3)	17 (16.0)	164 (31.2)
Parainfluenza virus 1, 2, 3, 4	51 (24.8)	53 (24.9)	18 (17.0)	122 (23.2)
Rhinovirus A/B/C	41 (19.9)	49 (23.0)	21 (19.8)	111 (21.1)
Influenza virus A and B	42 (20.4)	37 (17.4)	17 (16.0)	96 (18.3)
*Mycoplasma pneumoniae*	22 (10.7)	37 (17.4)	14 (13.2)	73 (13.9)
Adenovirus	32 (15.5)	27 (12.7)	9 (8.5)	68 (13.0)
*Streptococcus pneumoniae*	24 (11.7)	28 (13.1)	7 (6.6)	59 (11.2)
*Moraxella catarrhalis*	23 (11.2)	15 (7.0)	3 (2.8)	41 (7.8)
Metapneumovirus	23 (11.2)	9 (4.2)	5 (4.7)	37 (7.0)
*Bordetella pertussis*	12 (5.8)	7 (3.3)	6 (5.7)	25 (4.8)
*Haemophilus influenzae*	12 (5.8)	7 (3.3)	5 (4.7)	24 (4.6)
Coronavirus 229E/NL63 and OC43	6 (2.9)	7 (3.3)	2 (1.9)	15 (2.9)
*Staphylococcus aureus*	3 (1.4)	3 (1.4)	5 (4.7)	11 (2.1)
*Chlamydophila pneumoniae*	0 (0)	3 (1.4)	7 (6.6)	10 (1.9)
*Legionella pneumophila*	0 (0)	3 (1.4)	6 (5.7)	9 (1.7)
*Mycobacterium tuberculosis*	3 (1.4)	2 (0.9)	1 (0.9)	6 (1.1)
Enterovirus	2 (1.0)	2 (0.9)	2 (1.9)	6 (1.1)
Enterobacteriaceae^a^	3 (1.4)	2 (0.9)	1 (0.9)	6 (1.1)
*Coxiella burnetii*	3 (1.4)	2 (0.9)	0 (0)	5 (1.0)
Other Gram-positive bacteria^b^	2 (1.0)	1 (0.5)	0 (0)	3 (0.6)
Bocavirus 1/2/3/4	2 (1.0)	0 (0)	0 (0)	2 (0.4)
Gram-negative bacilli^c^	1 (0.5)	1 (0.5)	0 (0)	2 (0.4)
*Chlamydia trachomatis*	1 (0.5)	0 (0)	0 (0)	1 (0.2)
*Haemophilus parainfluenzae*	1 (0.5)	0 (0)	0 (0)	1 (0.2)
No identified microorganism	21 (10.2)	38 (17.8)	25 (23.6)	84 (16.0)
At least one virus	170 (82.5)	145 (68.1)	63 (59.4)	378 (72)
At least one pyogenic bacterium	65 (31.6)	61 (28.6)	23 (21.7)	149 (28.4)
At least one atypical bacterium	35 (17)	46 (21.6)	30 (28.3)	111 (21.1)

^a^ *Escherichia coli, Enterobacter cloacae*

^b^ *Enterococcus* spp.*, Streptococcus* spp.*, Staphylococcus epidermidis*

^c^ *Sphingomonas paucimobilis, Acinetobacter baumanii*

RSV and Metapneumovirus were especially frequent in children ≤2 years old, while most cases by *S. aureus*, *C. pneumoniae* and *L. pneumophila* occurred in children older than 5 years. All other viruses and *M. pneumoniae* had similar frequencies in all ages. Among children with CAP, 72% had at least one respiratory virus, 28% pyogenic bacteria and 21% atypical bacteria (Table 3). No organism was identified in 16% of children.

Among the 441 children and adolescents (84%) with respiratory pathogens identified, 172/441 (39%) had only one etiologic agent (121 children with virus, 25 with pyogenic bacteria, and 26 with atypical bacteria) and in 269/441 (61%) etiology was mixed (144 had 2 microorganisms; 79 had 3; 27 had 4; 13 had 5; 5 had 6, and one child had 7 different microorganisms). Combinations consisted of virus and pyogenic bacteria in 97 (97/441: 22%) cases; 81 (81/441: 18.4%) had two or more viruses; 58 (58/441: 13.1%) had virus and atypical bacteria; 15 (15/441: 3.4%) had virus with atypical and pyogenic bacteria; 9 had pyogenic and atypical bacteria; 6 had *M. tuberculosis* and other mixed infections; 2 had two or more atypical bacteria; and 1 had two or more pyogenic bacteria.

**Fig. 2:**
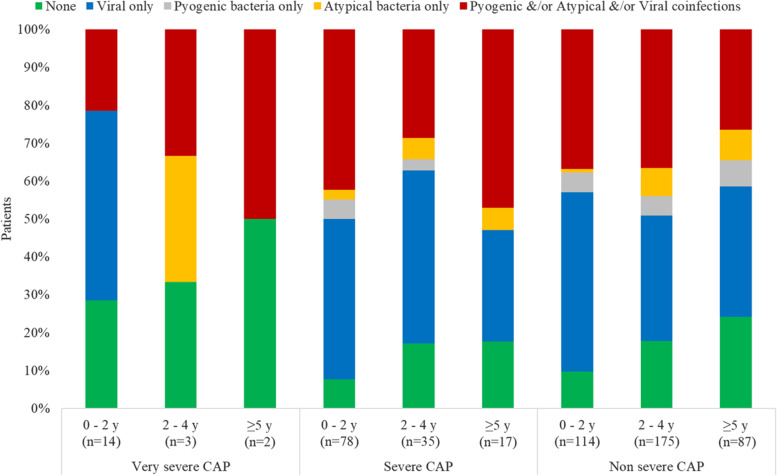
Percentage of microorganisms identified according to age and severity of community-acquired pneumonia. The figure shows the most common microorganisms identified in each category of severe community-acquired pneumonia (very severe, severe and non severe pneumonia) and by age group. Viral only, Pyogenic bacteria only, atypical bacteria only can have one or more microorganisms of the same group

Figure 2 shows the percentage of bacterial and viral infections identified according to age and severity of CAP. Among 19 children and adolescents with very severe pneumonia, RSV (*n* = 6/19), Rhinovirus (4/19) and Influenza (4/19) were the most common pathogens isolated. Among 130 children with severe pneumonia, RSV (49/130), Parainfluenza (28/130), Rhinovirus (27/130), and Influenza (22/130) were the most common pathogens isolated.

Around half of the control group had positive mPCR in tracheal aspirate for *S. pneumoniae* and *H. influenzae*. While *S. pneumoniae*, RSV, Rhinovirus, *M. pneumoniae* and Metapneumovirus were more frequently found in children with CAP, other microorganisms such as Adenovirus and *B. pertussis* were more common in the control group (Table 4). The frequency of Parainfluenza and Influenza virus was similar in both groups.

**Table 4:** Microorganisms detected in 525 children and adolescents with community acquired pneumonia and 61 controls, by mPCR, UA, IFA and acute serology IgM for *M. pneumoniae*

Microorganism, diagnostic test	CAP group (*N* = 525) n (%)	Control group (*N* = 61) n (%)
*Streptococcus pneumoniae:*		
mPCR	435 (82.9)	30 (49.2)
UA^a^	92 (17.9)	4 (8)
*Haemophilus influenzae*. mPCR	359 (68.4)	32 (52.5)
Respiratory syncytial virus A and B:		
IFA	39 (7.4)	4 (6.5)
mPCR	111 (21.1)	5 (8.2)
Parainfluenza virus 1, 2, 3, 4:		
IFA	33 (6.3)	6 (9.8)
mPCR	68 (12.9)	6 (9.8)
Rhinovirus A/B/C. mPCR	111 (21.1)	6 (9.8)
Influenza A and B:		
IFA	12 (2.3)	2 (3.3)
mPCR	47 (8.9)	5 (8.2)
*Mycoplasma pneumoniae*:		
mPCR	54 (10.3)	2 (3.3)
Acute serology IgM	170 (32.4)	23 (37.7)
Metapneumovirus. mPCR	37 (7.0)	0
Adenovirus:		
IFA	19 (3.6)	6 (9.8)
mPCR	4 (0.8)	0
Coronavirus 229E/NL63 and OC43. mPCR	15 (2.9)	0
*Bordetella pertussis*. mPCR	8 (1.5)	3 (4.9)
Enterovirus. mPCR	6 (1.6)^b^	0
*Chlamydophila pneumoniae.* mPCR	1 (0.2)	0
Bocavirus 1/2/3/4. mPCR	2 (0.5)^b^	0
*Legionella pneumophila:*	0	1 (1.6)
mPCR	0	1 (1.6)
UA	0	0

*IFA* indirect immunofluorescence assay, *UA* Urinary antigen, *mPCR* multiplex PCR

a UA: *n* = 515 in CAP group, and *n* = 50 in control group

^b^ mPCR for Enterovirus and Bocavirus: *n* = 379 in the CAP group and *n* = 61 in the control group (commercial diagnostic kit changed during the study to include these two new viruses, from RV12 to RV15 Seeplex® ACE Detection)

### Specimens and techniques to diagnose CAP

IS was collected in 94.7% of children with CAP, NS in 99%, urine samples in 98%, and paired serology in 93.9% (3.6% of the 525 children did not accept the second sample, and 2.5% were lost to follow-up). In addition, blood cultures were drawn in 78.3% (411/525), pleural fluid in 2.7%, gastric aspirate/lavage in 1.3%, and other samples in 9.3%. Among all IS samples, 465 had their microbiological characteristics available, and 339 (72.9%) were considered of good-intermediate quality; among them, microbiologic diagnosis by any method was reached in 114 children (33.6%). Blood cultures were positive in 3.6% (15/411), half of them with *S. pneumoniae*. All children in the control group had tracheal aspirate, blood and NS samples. *Eleven (*18%) had no urine sample available, and none had paired serology because the second blood sample was unavailable in this group.

Table 5 shows the contribution of different tests used for the etiological diagnosis in the CAP group. The mPCR was the main test to identify *M. pneumoniae* 74% (54/73), RSV 68% (111/164) and Parainfluenza virus 56% (68/122). mPCR (47/96: 49%) and paired serology (37/96: 37%) performed similarly for the detection of Influenza infection. Detection of *B. pertussis* (7/25: 28%), *C. pneumoniae* (1/10: 10%) and Adenovirus (4/68: 6%) was less accurate through mPCR (Table 5). *Legionella pneumophila* and *Coxiella burnetii* were only identified by paired serology, which also was the most important technique to diagnose *C. pneumoniae* (9/10: 90%) and Adenovirus 69% (47/68: 69%). Significant fourfold increase of antibody levels also occurred in Influenza (48/96: 50%), RSV (76/164: 46%), *M. pneumoniae* (33/73: 45%) and Parainfluenza (48/122: 39%). IFA yielded a diagnosis of less than 30% of respiratory viruses (Adenovirus 28%, Parainfluenza 27%, RSV 24% and Influenza 12%), but was the assay best suited for detection of *B. pertussis* (17/25: 68%).

**Table 5:** Comparative yield of positive microbiological results obtained for viruses and atypical bacteria in 525 children and adolescents with community acquired pneumonia

Microorganism, n (%)	mPCR	IFA	Paired Serology	IFA + mPCR	Paired Serology + mPCR	IFA + Paired Serology	IFA + mPCR + Paired Serology	Total cases of CAP attributed to each microorganism^a^
Respiratory Syncytial Virus A and B	64 (39)	11 (6.7)	37 (22.6)	13 (7.9)	24 (14.6)	5 (3)	10 (6.1)	164
Parainfluenza virus 1/2/3/4	48 (39.3)	22 (18)	30 (24.6)	4 (3.3)	11 (9)	2 (1.6)	5 (4.1)	122
Rhinovirus A/B/C	111 (100)	NA	NA	NA	NA	NA	NA	111
Influenza A and B	40 (41.7)	8 (8.3)	37 (38.6)	0 (0)	7 (7.3)	4 (4.2)	0 (0)	96
Adenovirus	2 (2.9)	19 (27.9)	45 (66.2)	0 (0)	2 (2.9)	0 (0)	0 (0)	68
Metapneumovirus	37 (100)	NA	NA	NA	NA	NA	NA	37
Coronavirus 229E/NL63and OC43	15 (100)	NA	NA	NA	NA	NA	NA	15
Enterovirus^b^	6 (100)	NA	NA	NA	NA	NA	NA	6
Bocavirus 1/2/3/4^b^	2 (100)	NA	NA	NA	NA	NA	NA	2
*Mycoplasma pneumoniae*	40 (54.8)	NA	19 (26)	NA	14 (19.2)	NA	NA	73
*Bordetella pertussis*	7 (28)	17 (68)	1 (4)	0 (0)	NA	NA	NA	25
*Chlamydophila pneumoniae*	1 (10)	NA	9 (90)	NA	0 (0)	NA	NA	10
*Legionella pneumophila*	0	NA	9 (100)	NA	0	NA	NA	9
*Coxiella burnetii*	NA	NA	5 (100)	NA	NA	NA	NA	5

*mPCR* multiplex PCR, *IFA* indirect immunofluorescence assay, *RSV* Respiratory Syncytial Virus, *NA* Not applicable

^a^Each column corresponds to the number of children with a positive result by the diagnostic techniques reported in the first row of the table (All columns are mutually exclusive)

^b^Enterovirus and Bocavirus 1/2/3/4 were studied in just 379 children because the first kit employed to detect respiratory viruses did not include these two ones

Seventy-six children (14.5%) were negative for *S. pneumoniae* by all available tests, and 390 (62.3%) were considered colonized (327 positive by mPCR in IS, 56 positive by mPCR and urinary antigen testing, and 7 positive only on urinary antigen testing). Fifty-nine (11.2%) children and adolescents had CAP which was attributable to *S. pneumoniae*, and among them IS culture had the highest diagnostic yield (35/59, 59.3%). Eight children (13.6%) with CAP attributable to pneumococcus had positive blood cultures (Table 6).

**Table 6:** Diagnostic yield (sensitivity) of different techniques used to detect 59 cases of community acquired pneumonia by *Streptococcus pneumoniae* (Sp) in 525 children and adolescents

mPCR in induced sputum *N* = 525	Culture in induced Sputum^a^ *N* = 357/465	Compatible Gram stain in good/intermediate induced sputum *N* = 339/465	Urinary antigen *n* = 514	Blood culture *N* = 411	Culture in Pleural effusion *N* = 14	Positive Results for *S. pneumoniae*
+	–	+	+	–	–	13
+	+	–	–	–	–	23
–	–	+	+	–	–	3
+	+	–	+	–	–	4
+	+	+	+	–	–	3
+	+	+	–	–	–	2
–	–	–	+	+	–	2
+	–	–	–	+	–	2
+	–	–	+	+	+	2
–	+	–	–	–	–	1
–	+	–	+	–	–	1
+	–	–	+	+	–	1
+	–	–	–	+	+	1
+	+	+	–	–	+	1
52 (88.1%)	35 (59.3%)	22 (37.3%)	29 (49.2%)	8 (13.6%)	4 (6.8%)	59

^a^Detection of *S. pneumoniae* by culture in induced sputum of good/intermediate quality with predominance of bacterial shapes compatibles with the Gram stain (gram-positive diplococci or gram-positive cocci)

CAP was attributed to *H. influenzae* in 24 children (4.6%) through isolation in good/intermediate quality IS (22 cases), in blood and cerebrospinal fluid in one child, and in another respiratory sample in the other case. *H. influenzae* was positive in IS by mPCR in the CAP group in 63.4% (333/525), versus 52.5% (32/61) in the control group. When mPCR was the only positive test for this bacterium, they were considered colonized.

## Discussion

Our study reports the incidence and etiology of CAP in requiring hospitalization in Colombian children and adolescents younger than 18 years old, and highlights the challenges faced when attempting to identify potential causative pathogens of CAP and the utility of different diagnostic tests. The incidence of pediatric CAP requiring hospitalization in our study was 147/100,000 (ranging from 608/100,000 in those younger than 2 years to 42/100,000 in those aged 5 to 17 years, respectively), consistent with other reports from Latin America. Gentile et al. [24] in a 2012 meta-analysis, estimated that the annual CAP incidence in Latin America was 919/100,000 in children younger than 5 years (1412/100,000 in those younger than 2 years and 539/100,000 between 2 to 5 years), and incidence rates in the United States in hospitalized children varied between 225/100,000 [25] and 157/100,000 [7]: 622/100,000 in those younger than 2 years, 238/100,000 from 2 to 4 years, 101/100,000 from 5 to 9 years, and 42 in 10–17 years [7].

Unlike previous Latin American studies investigating the etiology of pediatric CAP in the region [24, 26–31], including Colombian studies [32, 33], we identified at least one microorganism in 84% of hospitalized CAP patients, a percentage that is higher than other studies (ranging from 15.5 to 48%), and similar to the EPIC study (81%) [7] and Cevey-Macherel et al. (86%) [34]. The differences between studies may be explained by the use of IS, molecular tests and the combination of different tests, including microbiological stains, cultures and paired serologies [10, 11, 34]. Other factors that contribute to differences in reported observations included evaluation of different age groups, strict inclusion and exclusion criteria, and prospective versus retrospective study design [24, 28]. The historically reported low performance of several diagnostic tests and generally good outcomes have contributed to the low reliance of pediatricians on microbiological diagnosis of pneumonia in children [33].

One or more respiratory viruses were present in almost 3 of every 4 cases (72%), especially RSV, parainfluenza virus, rhinovirus, and influenza virus, similar to previous research [7, 9, 35, 36]. Comparing only the results of the mPCR because it was equally performed in case and control groups, only RSV and rhinovirus were more frequent in the CAP group compared to controls. In the EPIC study the attributable risks were 24.7% for RSV and 4.6% for rhinovirus (reported in 26 and 21.9% of pneumonia group and 1.9 and 17.3% of asymptomatic children, respectively), while other pathogens were only detected in less than 3% of asymptomatic group [9]. In addition, we found similar frequencies of parainfluenza, influenza, and adenovirus in the two groups. Therefore, the etiologic role of these viruses requires further study in our setting. A similar finding was reported by the EPIC study, that concluded that detections by real-time reverse-transcriptase PCR of parainfluenza, coronaviruses, rhinovirus, and adenovirus require further scrutiny [9].

It is worth highlighting the significant contribution of the mPCR used in our study for the diagnosis of *M. pneumoniae,* RSV and Parainfluenza virus, as well as IFA for the diagnosis of Adenovirus. However, less than 30% of respiratory viruses were diagnosed by IFA, one of the most common tests used in emergency rooms and hospitals. Caution when interpreting a positive test is required because of the limited knowledge of the duration of viable, replicating virus and the fact that many studies, including ours, have reported positive results in healthy children [9, 36].

As in many previous studies [10, 11, 27, 34, 37, 38], the frequencies of *S. pneumoniae* (11.2%) and *H. influenzae* (4.6%) in our study were higher than those found in the EPIC study [7], which reported a prevalence of 4 and 0.4%, respectively. In EPIC, this finding was attributed to a reduction in the burden of disease caused by these pathogens due to vaccination, the limited sensitivity of culture-based diagnostic tests, and the infrequent occurrence of bacteremic pneumococcal pneumonia. It is important to highlight three aspects that could explain the different results observed in comparison to the EPIC study [7]: i) IS was taken and cultured in all our patients (a specimen that better represents the content of the lower airway); ii) in our population, 61.8% of children under 2 years of age, and less than half of those over 2 years, had evidence of pneumococcal vaccination, regardless of the type of vaccine used, which contrasts with the almost universal use of the pneumococcal conjugate vaccine in the pediatric population in the United States; and iii) the percentage of the studied population that received antibiotics in the 72 h prior to hospitalization was lower (18% in the EPIC study vs 7% in ours), potentially contributing to the higher diagnostic yield of bacterial cultures in our study.

One of the most controversial aspects pertaining to the diagnosis of pneumococcal infection in the pediatric population is the use of urinary antigen, due to the apparent high rate of positivity reported in colonized children [39–42]. These studies have shown a good sensitivity of the test, but a limitation in specificity due to what is likely merely colonization of the upper airway in children. In the present study, according to positive mPCR in induced sputum, 82.9% of children with CAP were colonized, but only 17.9% had positive urinary antigen, while in the control group these percentages were lower (49.2 and 8% respectively). Similar frequencies of positive antigenuria in healthy children (between 3 and 8.7%) have also been reported in other countries [43]. Given the different rates of antigenuria found in colonized children, some have suggested that at least in settings with low rates of nasopharyngeal colonization, pneumococcal urine antigen testing may be useful for the diagnosis of pneumonia [41, 43–46]. Due to the aforementioned limitations of the assay, prospective studies are required to evaluate the usefulness of urinary antigen testing in children with and without CAP in different settings.

Collection of IS was a safe procedure in all children, regardless of age [20]. There were only two mild events of transient hypoxemia that improved when the procedure was temporarily interrupted, a similar finding reported by the PERCH study [47]. In some countries this procedure is only used for TB diagnosis in children, but not routinely done to investigate the etiology of CAP [48]. However, in our study, IS contributed to establishing the etiology of pneumonia in 33.6% of children, while other tests such as blood cultures had positivity of only 3.6%. Additionally, IS may increase detection of various pathogens, in addition to other specimens [36]. As the specimen originates from the most distal parts of the lung, IS better represents the content of the lower airway, and therefore, the microorganisms identified may play a pathogenic role in the CAP, compared to those detected in samples collected in the nasopharynx or oropharynx, which may only reflect upper respiratory tract colonization, or an asymptomatic infection [7].

Among the so-called atypical bacteria, *M. pneumoniae* was the most commonly identified in our study, occupying the fifth place in frequency (13.9%), versus the 8% reported by the EPIC study [7]. The proportion of cases attributed to *M. pneumoniae* varies widely depending on the geographic setting where the study is conducted and the technique used for diagnosis, ranging between 3.2 and 22% [10, 11, 34, 37]. Although the proportion of cases of *M. pneumoniae* by age group was similar, it is worth noting the high percentage observed in children of preschool age, different from the generalized concept that the proportion of cases of *M. pneumoniae* is higher as the age and level of schooling increases [7, 49, 50]. This finding cannot be explained by a selection bias or by a particular epidemic situation. On the other hand, because the positivity of IgM antibodies in the acute phase was similar in children with CAP and in the control group, this diagnostic test seems to provide little additional information [51]. Paired serology is not a practical tool for clinical diagnosis, while cultures are expensive and only done in reference centers, hence, molecular techniques have become the best option for the diagnosis of this bacterium [52]. Therefore, a combination of tests is often necessary in order to optimize the diagnosis [53], as was the case in our study, in which the simultaneous use of paired serology and mPCR in IS provided a higher diagnostic yield for CAP attributed to *M. pneumoniae*. The need to combine tests was also evidenced for the diagnosis of *C. pneumoniae* and *L. pneumophila*, which required almost exclusively paired serology due to the low sensitivity of the mPCR [54, 55]. It is necessary to evaluate new strategies to optimize the diagnosis of atypical bacteria in further studies [56].

It is important to highlight how the use of multiple diagnostic techniques allows the identification of an increased frequency of mixed infections. The most frequent combination was of viruses and pyogenic bacteria (18.5%), followed by two or more viruses (15.4%), and viruses with atypical bacteria (11.0%). In a cross-sectional study carried out in Argentina in 620 children under 6 years of age with acute respiratory infection, coinfection with 2 or more respiratory viruses was found in 12.8% of patients [57]. The EPIC study reported coinfections in 26% of children hospitalized for CAP, and demonstrated that children with typical bacteria, alone or in combination with respiratory viruses, have worse outcomes compared to those with a virus alone [58]. The clinical significance of mixed infection is uncertain [59], and the exact interaction between respiratory pathogens and their microbiota is unknown, albeit, some studies suggest synergistic relationships between pathogenic bacteria that are colonizers such as *S. pneumoniae, M. catarrhalis, H. influenzae,* or *S. pneumoniae* with respiratory viruses such as Influenza or RSV [60].

From a public health perspective, another important finding was the diagnosis of tuberculosis (TB) in 12 children and adolescents (2.3%) presenting with CAP, 6 confirmed through positive liquid culture in IS, and 6 unconfirmed TB. This result shows that TB may initially be indistinguishable from the most common pathogens of pediatric CAP, and therefore should always be considered in children who live in or visit TB endemic regions, especially when they fail to respond to standard antibiotic treatment; have risk factors to develop disease (for example, diabetes mellitus, present in one of our children diagnosed with TB); or present with recurrent lower respiratory infection (33% of our children diagnosed with TB had been hospitalized before for pneumonia, data not shown). In these cases, a more exhaustive evaluation is essential, focused on the epidemiological history, clinical evaluation, and close follow-up. This finding has been reported by other authors, who have also described cases of acute TB as a cause of pediatric pneumonia [10, 61], or lower respiratory infection [62]. TB has been reported to be an important contributor to pneumonia-related deaths in young children (4 to 20%) because of underdiagnosis or comorbidity predisposing to bacterial co-infection in TB-endemic settings [63]. Likewise, Moore et al. describe how pulmonary TB is frequently associated with severe *S. pneumoniae* pneumonia in African children and suggest that children hospitalized for CAP in this setting should be studied to exclude coexisting TB [61, 63].

The high number of techniques and diagnostic samples used, and the low percentage of lost-to follow-up observed during the study were some of our main strengths. However, we have some limitations: i) by excluding children with ≥ 72 h of antibiotics at hospital admission, we could have left out the most severe patients and generated a bias regarding to mortality and severity of the children included. However, as the objective of the study was focused on the etiology, our criteria for antibiotic duration had to be strict to avoid culture negativity and to give rigor to the investigation. Although we had no deaths in the study, during that year (2012) in Medellín 44 patients younger than 20 years did succumb to pneumonia; ii) our control group had a low number of children due to budget limitations, which also precluded the ability to assess paired serology in this group. Therefore, the percentage of healthy children who may have a four-fold rise in serological titers was not known; and iii) the incidence rates may have been underestimated due to the fact that children with CAP were not recruited in all the city’s hospitals (although our study covered most of them, and included those that serve the largest number of patients).

## Conclusions

The combination of multiple specimens and different laboratory techniques, as well as adequate interpretation of the results obtained, is required in order to attain a correct identification of the etiology of pneumonia**,** especially in children and adolescents. Viruses, mainly RSV, continue to be the most common cause of CAP in children requiring hospitalization, especially in those under 5 years of age. *S. pneumoniae* was ranked seventh as an etiologic agent of child and adolescent CAP in our study. Induced sputum is a useful specimen**,** and **is** safe for the etiological diagnosis of CAP in children and adolescents (following rigorous sample collection and processing protocols). Tuberculosis should also be considered as a cause of CAP in TB endemic countries and patients with risk factors. Future studies should evaluate the role of mixed infections in CAP, improve techniques for early and accurate identification of *M. pneumoniae*, with the ability to elucidate the role which that organism plays as a pathogen versus a colonizer detected in the respiratory tract of these patients.

## Supplementary Information

**Additional file 1:** **Appendix 1.** Microbiological definitions used in the study.

## Data Availability

The datasets used and analyzed during the current study are available upon request through the corresponding author.

## Abbreviations

CAP: Community-acquired pneumonia
IS: Induced sputum
NS: Nasopharyngeal swab
mPCR: Multiplex PCR
IFA: Indirect immunofluorescence assay
RSV: Respiratory Syncytial Virus
