# Supplementary material for: Etiology and the challenge of diagnostic testing of community-acquired pneumonia in children and adolescents
Source: BMC Pediatr. 2022 Mar 31;22:169. doi: 10.1186/s12887-022-03235-z (PMC8968093; doi:10.1186/s12887-022-03235-z)
Supplement: Supplementary file 1 — Additional file 1: Appendix 1. Microbiological definitions used in the study. [file 12887_2022_3235_MOESM1_ESM.docx]

Appendix 1. Microbiological definitions used in the study.

- *Streptococcus pneumoniae* (at least one of the following): isolation in cultures of sterile sites; IS with less than 25 SECs/LPF showing dominance of compatible forms ( Gram positive cocci or diplococci) with or without isolation of *S. pneumoniae*; and/or a positive urine antigen accompanied by a culture or Gram of IS compatible with this bacterium.
- *Haemophilus influenzae*: isolation in cultures of sterile sites; or IS with less than 25 SECs/LPF showing dominance of compatible forms (Gram negative coccobacilli), with or without isolation of *H. influenzae*.
- Other bacteria (*Moraxella catarrhalis*, *Bordetella pertussis*, *Staphylococcus aureus*, enterobacterias, other gram-positive cocci and other gram-negative bacilli): isolation of a microorganism known to cause pneumonia in IS culture (correlating with Gram stain in a good quality sample); or isolation in sterile sites (i.e., cerebrospinal fluid, pleural fluid or blood) with no other explanation.
- Virus: detection of viruses in nasopharyngeal swabs by immunofluorescence, fourfold increase in IgG antibody titers in paired serum specimens, or positive mPCR in IS samples.
- *B. pertussis***:** antigen detection in nasopharyngeal swabs by direct immunofluorescence, or positive mPCR in IS samples.
- *M. pneumoniae:* fourfold increase in IgM or IgG antibody titers in paired serum specimens, or positive mPCR in IS samples.
- *C. pneumoniae:* fourfold increase in IgM or IgG antibody titers in paired serum specimens, or positive mPCR in IS samples.
- *C. burnetii:* fourfold increase in IgM or IgG antibody titers in paired serum specimens.
- *L. pneumophila:* fourfold increase in whole antibody titers in paired serum specimens, positive urine antigen, or positive mPCR in IS samples.
- Tuberculosis (TB): isolation of *M. tuberculosis* in culture of IS or gastric aspirate. Patients were considered suspicious of pulmonary TB when they had at least 2 of the following: close TB contact, positive tuberculin skin test (≥10 mm), pulmonary abnormalities consistent with TB on chest X-rays and clinical response to anti-tuberculosis therapy. The cases were classified according to the latest consensus of intrathoracic TB, as confirmed TB and unconfirmed TB [61].
